# Magnetoencephalography (MEG) Data Processing in Epilepsy Patients with Implanted Responsive Neurostimulation (RNS) Devices

**DOI:** 10.3390/brainsci14020173

**Published:** 2024-02-09

**Authors:** Pegah Askari, Natascha Cardoso da Fonseca, Tyrell Pruitt, Joseph A. Maldjian, Sasha Alick-Lindstrom, Elizabeth M. Davenport

**Affiliations:** 1 Radiology Department, The University of Texas Southwestern Medical Center, Dallas, TX 75390, USA; pegah.askari@utsouthwestern.edu (P.A.); natascha.cardosodafonseca@utsouthwestern.edu (N.C.d.F.); tyrell.pruitt@utsouthwestern.edu (T.P.); joseph.maldjian@utsouthwestern.edu (J.A.M.); sasha.alick-lindstrom@utsouthwestern.edu (S.A.-L.); 2 MEG Center of Excellence, The University of Texas Southwestern Medical Center, Dallas, TX 75390, USA; 3 Biomedical Engineering Department, The University of Texas Southwestern Medical Center, Dallas, TX 75390, USA; 4 Biomedical Engineering Department, The University of Texas at Arlington, Arlington, TX 76010, USA; 5 Neurology Department, The University of Texas Southwestern Medical Center, Dallas, TX 75390, USA

**Keywords:** responsive neurostimulation (RNS), magnetoencephalography (MEG), drug-resistant epilepsy (DRE), independent component analysis (ICA), ICA-based signal processing, MNE-python

## Abstract

Drug-resistant epilepsy (DRE) is often treated with surgery or neuromodulation. Specifically, responsive neurostimulation (RNS) is a widely used therapy that is programmed to detect abnormal brain activity and intervene with tailored stimulation. Despite the success of RNS, some patients require further interventions. However, having an RNS device in situ is a hindrance to the performance of neuroimaging techniques. Magnetoencephalography (MEG), a non-invasive neurophysiologic and functional imaging technique, aids epilepsy assessment and surgery planning. MEG performed post-RNS is complicated by signal distortions. This study proposes an independent component analysis (ICA)-based approach to enhance MEG signal quality, facilitating improved assessment for epilepsy patients with implanted RNS devices. Three epilepsy patients, two with RNS implants and one without, underwent MEG scans. Preprocessing included temporal signal space separation (tSSS) and an automated ICA-based approach with MNE-Python. Power spectral density (PSD) and signal-to-noise ratio (SNR) were analyzed, and MEG dipole analysis was conducted using single equivalent current dipole (SECD) modeling. The ICA-based noise removal preprocessing method substantially improved the signal-to-noise ratio (SNR) for MEG data from epilepsy patients with implanted RNS devices. Qualitative assessment confirmed enhanced signal readability and improved MEG dipole analysis. ICA-based processing markedly enhanced MEG data quality in RNS patients, emphasizing its clinical relevance.

## 1. Introduction

Epilepsy is a chronic neurological disorder characterized by recurrent, unprovoked seizures resulting from abnormal electrical activity in the brain [1]. While anti-seizure medication (ASM) can effectively control seizures in most cases, approximately 30% of patients experience drug-resistant epilepsy (DRE) [2]. This subset, failing to achieve seizure control despite appropriate ASM treatment presents a significant clinical challenge [3,4]. Surgical intervention, aimed at accurately localizing and addressing the epileptogenic zone(s), is an efficacious treatment for DRE [3,4,5]. The primary goal of epilepsy surgery is the complete resection or disconnection of the epileptogenic zone while preserving eloquent cortices, emphasizing the importance of precise localization and intervention [6].

An adjuvant therapy for DRE patients is neuromodulation, including FDA-approved devices such as vagus nerve stimulation (VNS) [7], deep brain stimulation (DBS), and responsive neurostimulation (RNS). The RNS^®^ System is an FDA-approved implantable responsive neurostimulator device which is indicated when there are no more than two epileptogenic foci, or with seizures originating from eloquent cortices, which cannot be resected. The RNS device (RNS^®^ System; NeuroPace, Inc., Mountain View, CA, USA) continuously monitors electrocorticographic (ECoG) activity and delivers targeted electrical stimulation to suppress seizures [8]. After extensive pre-surgical evaluation to identify the seizure onset zone (SOZ), including stereo electroencephalography (SEEG), the RNS electrodes are placed in the area determined to be the SOZ. The RNS electrodes detect the abnormal electrical patterns associated with seizure onset, and the device promptly provides stimulation to disrupt the seizure cascade, preventing the occurrence of clinical and electrographic seizures. RNS offers a personalized treatment modality for DRE patients, delivering real-time intervention tailored to their specific seizure patterns [1].

While the RNS system may provide significant benefits in managing DRE patients, some patients may still experience persistent seizures or develop new seizure foci. Magnetoencephalography (MEG), a valuable FDA-approved, noninvasive technique, plays a crucial role in the preoperative evaluation of DRE patients and/or evaluation in cases warranting reoperation [9]. Utilizing superconducting quantum interference devices (SQUIDs), MEG measures the brain’s magnetic fields, ranging from femto-tesla to pico-tesla [10,11], with millisecond precision and a spatial resolution of 2–3 mm [12]. MEG has proven valuable in mapping functional brain networks, localizing epileptic seizure foci, and aiding in pre-surgical planning [13]. MEG can also help identify eloquent cortices, which is important in order to minimize functional deficits during epilepsy surgery [11].

The use of MEG for surgical planning in operative re-interventions, particularly in patients experiencing ongoing seizures after RNS system placement, has been limited. This is largely due to the assumption that electric and magnetic interference from the RNS device [10,14] would lead to a non-diagnostic study. Many services refrain from attempting MEG scans in RNS patients due to these challenges, further emphasizing the need for innovative solutions to integrate MEG into standard care for these patients. MEG is critical for understanding brain activity dynamics, seizure initiation, and the impact of RNS on neural networks. Techniques like temporal signal space separation (tSSS) [14,15] have been explored to mitigate signal distortion but have not consistently yielded satisfactory results. The limited effectiveness of current strategies underscores the urgency for user-friendly and automated methods to augment MEG’s clinical utility in RNS patients and make the noninvasive approach more practical and efficient. Our aim is to address the challenges posed by the RNS neurostimulator [10], and to fill this critical gap in MEG clinical practice. This advancement is essential for enhancing our understanding of post-RNS brain function and improving targeted treatments for improved seizure control and patient outcomes.

It is important to effectively reduce the contamination caused by environmental noise, sensor noise, and physiological artifacts while retaining the signal quality so that it enhances the accuracy and reliability of the analysis of brain activity. Incorrectly applying artifact rejection methods could unintentionally eliminate sources of interest or lead to erroneously interpreting overmanipulated or poorly manipulated data as abnormal brain activity, making the choice and implementation of artifact rejection methods critical. There are several artifact rejection methods commonly used in MEG. These methods aim to identify and remove various types of artifacts to enhance the quality of MEG data. Some of these artifact rejection techniques include tSSS [15] and independent component analysis (ICA) [16,17]. tSSS constructs a subspace based on spatially coherent artifacts captured by reference sensors and projects this subspace out of the MEG data, effectively removing artifact contamination and improving the accuracy of neural activity analysis [15]. The tSSS method requires only two assumptions: the brain and external interference sources can be separated geometrically, and the brain signals are not temporally correlated with any signal from a nearby artifact source [18]. In this way, tSSS removes the substantial interference caused by external and nearby sources. The tSSS method has been shown to be effective for noisy datasets contaminated by contributions from dental braces and fillings, metal implants, or stimulators [19]. ICA is a technique for estimating independent source signals from recordings in which the source signals were mixed in unspecified ratios. Typically, users are required to select components representing artifacts; however, recent advancements, such as those in MNE-Python (package version 0.23.4), an open-source software for MEG signal processing, have automated this procedure [16]. This function decomposes the temporal data into a predetermined number of components, from which either an algorithm or a user with expertise identifies and designates the components that represent noise, and these are projected out of the temporal data. This process entails the removal of the identified noise-afflicted components from the temporal data domain. Other artifact rejection techniques include automatic bad channel detection, trial rejection based on amplitude thresholds, and artifact subspace reconstruction [20]. Specifically, the “ft_rejectartifact” function in Fieldtrip enables the semiautomatic detection of well-defined artifacts such as eye blinks, muscle contractions, or MEG SQUID jumps [20]. The peak-to-peak amplitude rejection method involves rejecting MEG trials that exhibit excessive peak-to-peak amplitudes exceeding a predefined threshold [21]. It is most effective in identifying and removing artifacts caused by sudden movements, muscle activity, or environmental interference [21]. It is important to note that different artifact rejection methods may suit different types of artifacts. Therefore, combining multiple techniques is often employed to achieve robust artifact removal in MEG data analysis.

In the context of addressing the specific challenge of signal separation in MEG recordings of patients with implanted RNS, this study innovates by applying ICA subsequent to the conventional tSSS method. Employing ICA within an automated framework facilitates the identification and removal of artifacts, significantly enhancing the robustness and precision of MEG data analysis by effectively excluding noise components while preserving the underlying neural activity. Furthermore, to optimize the efficiency of this challenge, we propose the automated ICA approach using MNE-Python, a powerful open-source software package for MEG and electroencephalography (EEG) analysis [16]. This novel application of a well-established technique, ICA, in the context of clinical MEG analysis for RNS patients, not only streamlines the data analysis process but also significantly enhances the quality, consistency, and objectivity of the analysis. MNE-Python provides an added advantage by eliminating the need for costly proprietary software such as MATLAB, making it a cost-effective solution. Additionally, the automated nature of the approach allows it to be easily implemented. By applying automated ICA-based noise reduction techniques, we anticipate substantial improvements in the quality and reliability of MEG data obtained from patients with RNS devices. It represents a significant advancement in the field, providing clinicians with a robust, efficient, and objective method for analyzing MEG data in this unique patient population.

## 2. Materials and Methods

### 2.1. Participants

The MEG scans, performed as part of standard clinical care, utilized a 306-channel MEGIN Triux-Neo MEG system and were acquired concurrently with EEG data. Clinical resting-state MEG signals from two epilepsy patients with implanted RNS devices and one epilepsy patient without an implanted RNS device are presented in this study. Patient 1 and Patient 2, a 42-year-old female and a 30-year-old male, respectively, were considered DRE patients due to ongoing seizures despite adequate trials of two tolerated and appropriately chosen ASMs [4]. Patient 1 had frontal dysplasia and lesional focal frontal lobe epilepsy overlapping with the supplementary motor area (SMA). Patient 2 had non-lesional multifocal epilepsy, with SOZ overlapping with Broca’s area. Patient 2 had a previous, limited frontal lobe resection that spared Broca’s area prior to MEG scan. As the SOZ overlapped with eloquent cortical regions in both patients, a recommendation was made for RNS device placement. However, despite undergoing RNS device placement and programming over time, both patients continued to experience seizures. The RNS devices for both patients were NeuroPace (RNS-320) (Mountain View, CA, USA). Both patients had RNS devices placed at outside hospitals; therefore, limited information was available regarding the presurgical workup. However, chart review of the presurgical workup revealed that MEG was not performed in these particular cases. Unlike RNS-300M models of the RNS neurostimulator, which are magnetic resonance imaging (MRI)-unsafe, individuals with the RNS neurostimulator model RNS-320, classified as MRI-conditional, are able to undergo MRI and MEG procedures. The RNS device location on Patient 1 was at the left occipital/parietal region with strip electrodes in the left SMA. The RNS device location on Patient 2 was at the left frontal region with strip electrodes in the left frontal region. The time from RNS placement to the MEG scan was 3 and 4 years for patients 1 and 2, respectively. Patient 3 was a 67-year-old male without implanted RNS or other devices. Detailed information for each patient is available in Table A1.

### 2.2. Resting-State Protocol and MEG Data Acquisition

The MEG data were acquired while the participant was supine in a magnetically shielded room (MSR). MEG recordings were collected continuously at a 1000 Hz sampling rate with a system that includes 204 planar gradiometers and 102 magnetometers. Participants were sleep deprived prior to the scan and, during the scan, were asked to keep their eyes closed and fall asleep if possible. The duration of these recordings was about 60 min. Electrooculogram (EOG) and electrocardiogram (ECG) electrodes were used to record the vertical and horizontal eye movements, and heartbeat artifacts, respectively. In addition, the head position indicator (HPI) coils were attached to the scalp and measured head position at the start and end of each scan. A FASTRAK digitizer was used to record locations of the HPI coils as well as three anatomical fiducials for the nasion, left and right pre-auricular points (LPA and RPA, respectively), and approximately 500 points across the scalp to help with the co-registration of MEG data with their corresponding T1-weighted MRI.

### 2.3. RNS Device Settings during MEG Scan

The RNS device can be programmed to monitor and deliver responsive stimulation to no more than two epileptogenic foci. When a specific ECoG pattern is detected, up to five individually configured sequential stimulations can be delivered. Each stimulation can contain two bursts that can be independently configured. The RNS device can also store segments of electrocorticographic activity, neurostimulator status indicators, and records of events detected by the neurostimulator [22].

Clinicians can program how the neurostimulator operates [23]. In this study, RNS was recorded in two modes, an MRI mode and an off mode [10]. The MRI mode disables the stimulation, but enables detection and storage. This means that the RNS device cannot stimulate the brain but still monitors and records the brain signal, looking for changes and stores them. The off mode disables the stimulation, detection, and storage. Off mode helps to avoid the limited recording utility of the MEG due to the artifact of the RNS electronics.

### 2.4. Standard Clinical Preprocessing (Conventional Preprocessing)

Standard clinical pre-processing, tSSS, was performed on raw MEG data using the scanner manufacturer’s software MaxFilter (version 2.2.14—US). The patient’s center of sphere was calculated using Mrilab (version 1.7.25). These patient-specific coordinates, a tSSS subspace correlation of 0.88, and a tSSS buffer length of 4 s were utilized in MaxFilter. All other parameters were set to the manufacturer default.

### 2.5. Automated ICA (ICA-Based Noise Removal Preprocessing)

**Figure 1:**
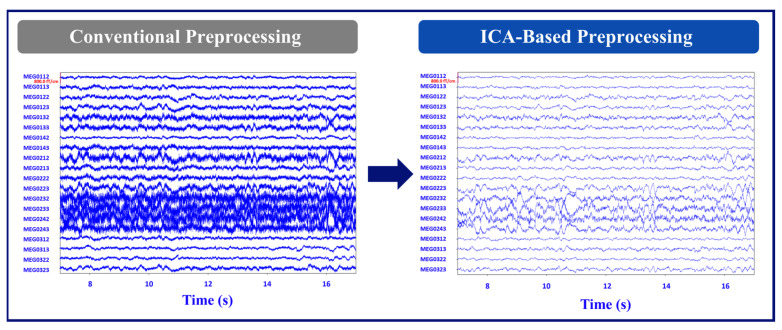
Example of conventional vs. ICA-based preprocessing of time series data. This illustrates 20 s of MEG signal data from 20 MEG channels out of 306 channels for the conventional vs. ICA-based preprocessing methods (Patient 1). The plot on the left shows the MEG signal with the conventional preprocessing, which includes tSSS only. The plot on the right shows a cleaner signal after the ICA-based preprocessing steps were performed on the data after tSSS.

MEG sensor-level data were then further pre-processed using the freely available MNE-Python package [16]. First, a 1–70 Hz band-pass filter was applied to the tSSS resting-state file. The default window method was used for the finite impulse response (FIR) filter design. Then, ICA was performed using a pre-defined rejection band (magnetometer = 5 × 10^−12^ T, gradiometer = 4 × 10^−10^ T) to avoid fitting ICA in situations with significant environmental artifacts. We used 25 components for fitting ICA, and the ICA method was set to the default “fastica” method. The FastICA estimation is non-deterministic, so fixing the seed to have reproducible results is practical. To this end, the random state was set to 23. The EOG and ECG artifacts were automatically removed using ICA. EOG and ECG recordings were available, so it automatically selected the corresponding artifact components from the decomposition. Finally, the rejected components were removed by the application of the ICA function, and saved into a new FIF file for evaluation with MEGIN clinical software (Graph version 2.94). Figure 1 illustrates the MEG signal after the conventional and ICA-based noise removal preprocessing.

### 2.6. Power Spectral Density and Signal-to-Noise Ratio Calculation

The power spectral density (PSD) was computed using Welch’s method. First, the average PSD from all channels was calculated for both processing methods in dB, and the values were used to calculate and compare the signal-to-noise (SNR) ratio from conventional and ICA-based preprocessing steps. Secondly, for both preprocessing methods, the SNR was calculated and compared for channels directly above the RNS neurostimulator and channels on the contralateral hemisphere with less RNS interference in Patients 1 and 2. For Patient 3, we adopted the noise and signal hemispheres identical to those of Patient 1 for the purpose of SNR and PSD calculations. Finally, statistical analysis was reported in terms of “mean ± standard deviation” in decibels (mean ± SD).

**Figure 2:**
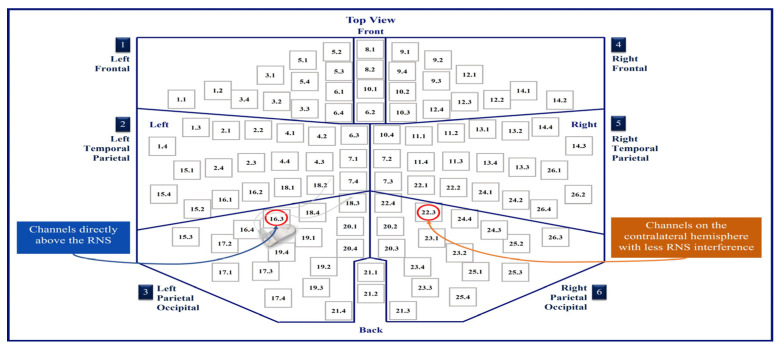
MEG sensor layout. This illustrates the MEG sensor layout and the RNS device location for Patient 1. The RNS device was located in the left occipital/parietal region.

**Figure 3:**
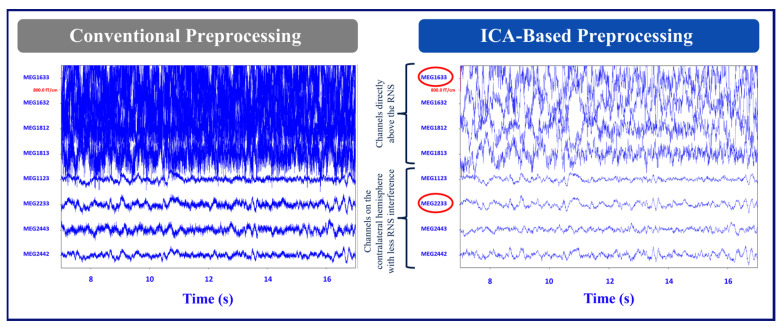
Example time series data. This illustrates 20 s of MEG signal data from 8 MEG channels out of 306 channels for the conventional vs. ICA-based preprocessing methods (Patient 1). The top four channels are the MEG channels directly above the RNS, and the bottom four are the MEG channels on the contralateral hemisphere with less RNS interference.

**Figure 4:**
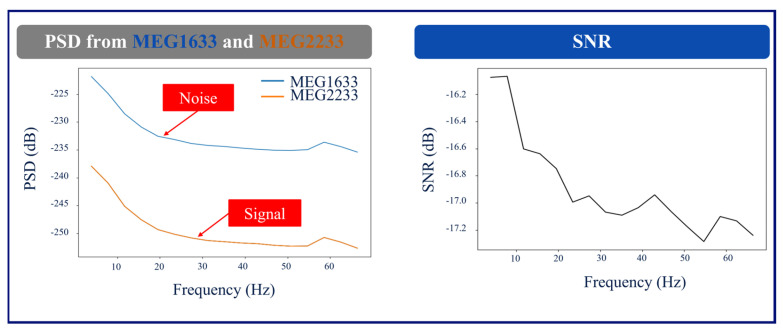
Example PSD and SNR Plots. The plots of PSD and SNR resulted from MEG1633 (Noise) and MEG2233 (Signal) for Patient 1. MEG1633 is located above the RNS, which was considered to be the noise, and MEG2233 is located on the contralateral hemisphere of the brain from the RNS, which was considered to be the signal.

As an illustration, Figure 2 shows the layout of the MEG channels along with the location of the placement of the RNS device in Patient 1 (left occipital/parietal region). In this patient, the channels on the left parietal/occipital region are the channels above the RNS device, and the channels on the right parietal/occipital region are the channels on the contralateral hemisphere with less RNS interference. Figure 3 shows the conventional and ICA-based preprocessing of MEG signals in channels above the RNS device versus the channels on the contralateral side of the hemisphere from the RNS device for the same patient. Figure 4 shows a graph of the PSD and SNR calculations corresponding to MEG1633 and MEG2233 channels after ICA-based preprocessing.

### 2.7. MEG Dipole Analysis

The MEG data underwent analysis by a board-certified clinical neurophysiologist with additional training in clinical MEG. The source estimation of the visually detected spikes was performed using the single equivalent current dipole (SECD) model in xfit (clinically approved software from MEGIN (xfit version 5.5)). During the assessment of the SECD fitting, the following statistical criteria were employed: a goodness of fit > 80%, a confidence volume of <1000 mm^3^, a reduced chi-square value of 0.5–2.0, and a dipole moment ranging from 100–500 nAm. The analysis was performed using data that underwent conventional and ICA-based preprocessing.

Dipole fitting with both conventional and ICA-based analyses were performed prior to a potential second intracranial study or resection. Results were interpreted in the context of all clinical data and ancillary testing results. As part of their presurgical and postsurgical evaluation at our high-volume NAEC (National Association of Epilepsy Centers) level 4 epilepsy center, patients were presented at a multidisciplinary epilepsy team conference, including epileptologists, neurosurgeons, neuroradiologists, psychiatrists, and psychologists. The MEG dipole data from the conventional processing was presented and discussed along with other data, including seizure clinical semiology and electroclinical findings, in addition to a battery of noninvasive and invasive results. At the conclusion of these patient management conferences, a hypothesis of the SOZ was formulated.

The analysis of the preprocessed MEG data with ICA-based preprocessing method was performed as part of the research protocol and was not related to the epilepsy team’s plan recommendation. The primary aim being to assess the viability of the processed data for dipole modeling with acceptable statistical criteria. However, the results of the ICA-based processing were discussed with a subset of the multidisciplinary team, in a similar manner to the multidisciplinary team conference.

## 3. Results

### 3.1. Signal-to-Noise Ratio Analysis

The recordings taken while the RNS devices were in MRI mode had extremely limited recording quality that resulted in multiple errors and failures in MaxFilter when attempting to apply tSSS. Therefore, the SNR values from the RNS devices in MRI mode are not reported. The SNR values from patients 1 and 2 with RNS devices in off mode, as well as Patient 3, without an RNS device, are shown in Table 1. The first row of data in Table 1 shows the SNR mean and standard deviation (SD) calculated for the conventional versus the ICA-based method. A better signal improvement was seen in Patient 1. The second and third row of data in Table 1 shows the SNR for the conventional and ICA-based methods, respectively, assessing the channels above the RNS devices versus the channels on the contralateral hemisphere of the brain for Patients 1 and 2. We evaluated the SNR in Patient 3 using the same methodology and hemisphere selection as we did for Patient 1. The SNR improved in both RNS patients in the ICA-based method.

Negative SNR values in Table 1 imply that the noise is much greater than the signal where the RNS device is located in patients 1 and 2. In addition to these quantitative findings, a board-certified epileptologist qualitatively confirmed the improved readability of the signals after ICA-based preprocessing.

### 3.2. MEG Dipole Analysis and Comparison with Clinical Results

With the ICA-based preprocessing method, we were able to model all previously modeled dipoles, as well as to add more dipoles to represent time points that were previously unable to be modeled with acceptable statistical criteria.

Patient 1, with seizure onset at age ten months, had an MRI showing extensive left superior and medial frontal lobe cortical dysplasia. The patient underwent SEEG, as well as grid placement, at an outside hospital that identified a probable SOZ, including dysplasia in the left frontal lobe and SMA. The patient also had an interictal single-photon emission-computed tomography (SPECT) but not SISCOM (subtraction ictal SPECT co-registered to MRI) demonstrating decreased perfusion in the left frontal lobe and neuropsychological evaluation demonstrating dysfunction in frontal/subcortical cerebral networks. The outside hospital recommended resection, which the patient declined due to concerns for potential postop deficits. The patient underwent RNS device placement in the left frontal lobe at the outside hospital. However, her seizures persisted and she was referred to our institution for further evaluation, including a MEG scan, for possible surgical options. Following repeat testing, the epilepsy team recommended RNS device removal with resection of the SMA and frontal region dysplasia with the reimplantation of the RNS device.

**Figure 5:**
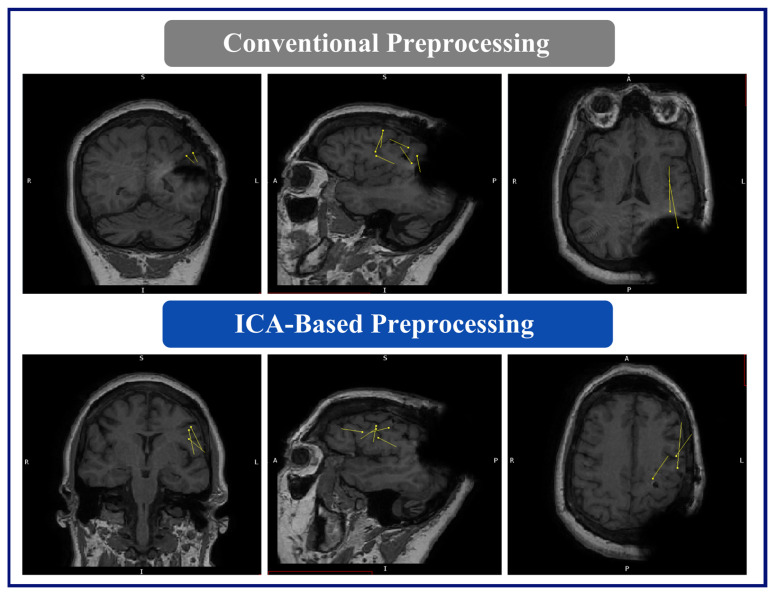
MEG dipole analysis: A comparison of dipole analysis between conventional and ICA-based preprocessing techniques on Patient 1. This figure illustrates that the dipoles (represented by yellow lines and triangles) fitted with the ICA-based preprocessed data show diminished association with the RNS device artifact area and increased concordance with the hypothesized seizure onset zone (left inferior frontal and precentral gyri) compared to conventional preprocessing.

The MEG results for this patient were as follows: (A) Conventional preprocessing—tight clusters in the left precentral and postcentral gyri, the latter bordering the large artifact area, and a loose cluster in the left anterior insula, inferior frontal operculum, and middle frontal gyri. (B) ICA-based preprocessing—a tight cluster in the left inferior frontal gyrus and precentral gyrus, a loose cluster in the insula and Rolandic operculum, and isolated dipoles in the cingulate gyrus, the SMA, and the middle and superior frontal gyri within, or bordering on, the dysplastic area (Figure 5).

Patient 2, with seizure onset at eight years old, had a previous SDE (subdural evaluation) which showed left basal frontal, inferior frontal, anterior temporal, inferior temporal, and insular seizure onset zones. After intracranial evaluation, the fMRI evidenced the overlapping of Broca’s area with epileptogenic focus and a Wada test suggested left language lateralization. The patient underwent left frontal disconnection procedures due to proximity to Broca’s area, and responsive neurostimulator placement.

Despite numerous interventions, as described, his seizures persisted. The MRI evidenced generalized atrophy of the left temporal lobe, the left frontal resection, and the RNS device placement. Post-operative positron emission tomography (PET) suggested epileptogenic focus in the left frontal lobe posterior to the resection cavity. Ictal SPECT demonstrated increased perfusion posterior to the left frontal resection with reduced interictal and neuropsychological evaluation evidencing frontal/subcortical networks of the dominant language hemisphere.

**Figure 6:**
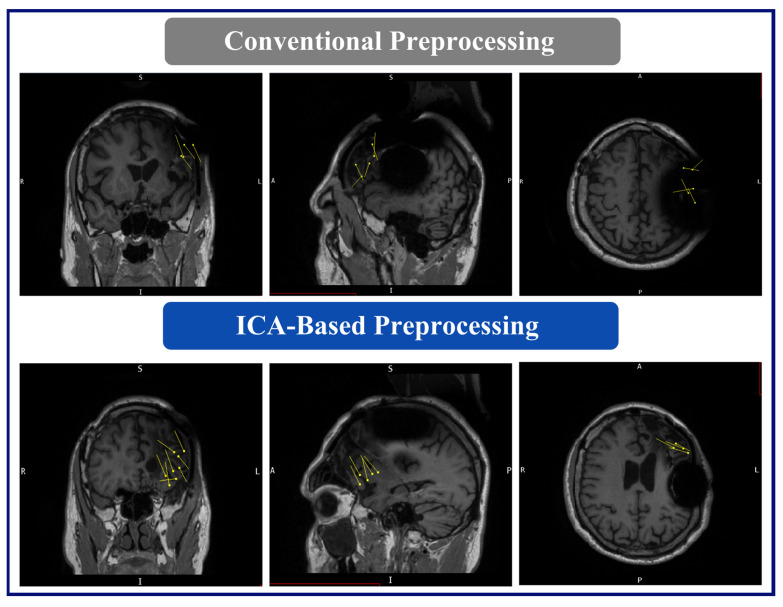
MEG dipole analysis: A comparison of dipole analysis between conventional and ICA-based preprocessing techniques on Patient 2. This figure also illustrates that the dipoles (represented by yellow lines and triangles) fitted with the ICA-based preprocessed data show diminished association with the RNS device artifact area and increased concordance with the hypothesized seizure onset zone (left inferior frontal gyrus and left insula) compared to the conventional preprocessing, indicating enhanced localization and reduced interference.

The MEG results for this patient were as follows: (A) Conventional preprocessing—A loose cluster in the left inferior and middle frontal gyri in the expected region of Broca’s area, and seven additional dipoles, which were mapped within the large artifact area in the left frontal lobe. (B) ICA-based preprocessing—Two tight clusters, one in the left inferior frontal gyrus posterior to the resection area, and one in the nearby anterior insula, with two additional dipoles within the left middle frontal gyrus, posterior to the resection (Figure 6).

The patient and family were not interested in a new invasive EEG evaluation or resection. The interdisciplinary epilepsy surgical committee discussed the case and agreed that the patient could benefit from adding centromedian DBS therapy to existing RNS therapy.

**Figure A1:**
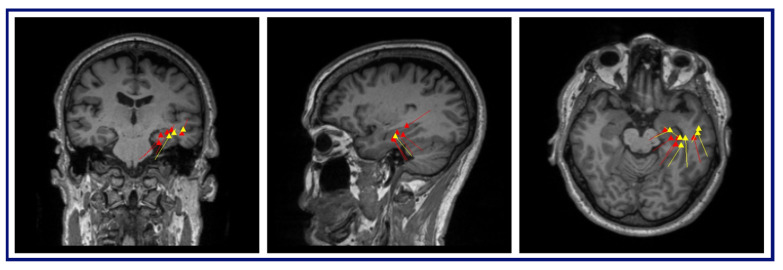
MEG dipole analysis: A comparison of dipole analysis between conventional and ICA-based preprocessing techniques on Patient 3. Yellow dipoles illustrate the conventional preprocessing and red dipoles illustrate the ICA-based preprocessing. This figure illustrates that the dipoles fitted with the ICA-based processed data indicate the same localization as conventional preprocessed data in a patient without RNS.

The dipoles were localized to the same location as the conventional processing used for Patient 3 (Figure A1).

## 4. Discussion

The dipoles localized using the MEG data with the RNS device in off mode and ICA-based noise removal preprocessing analysis were in areas closer to the probable seizure onset zone and aligned with the treatment recommendations and surgical plan more than the traditional preprocessing method.

SNR comparisons between the conventional and ICA-based methods revealed that the ICA-based method exhibited superior signal improvement in both patients. Particularly, Patient 1 showed significantly better results with the ICA-based method. The SNR values demonstrated consistent improvements when assessing channels above the RNS device compared to channels on the contralateral hemisphere of the brain for both patients with RNS. These findings suggest the effectiveness of the ICA-based method in enhancing SNR and highlight its potential benefits for improving RNS recordings in off mode.

Dipoles fit with the ICA-based processed data demonstrated a reduced relationship with the RNS artifact area, i.e., the clusters and dipoles localizing to areas well-distinguished and further from the area of the RNS device, demonstrating less interference from the artifact. In the case of Patient 1, the conventional preprocessing showed tight clusters in the left precentral and postcentral gyri, bordering an area on the MRI showing a large artifact from the RNS device. However, with the ICA-based preprocessing, the tight cluster was found within the left inferior frontal and precentral gyri, with isolated dipoles localizing to the SMA, and bordering or within the dysplastic frontal lobe area, suggesting a more accurate localization within the clinically relevant regions of interest. Similarly, in the case of Patient 2, the conventional preprocessing showed dipoles bordering or within the large artifact area in the left frontal lobe. In contrast, the ICA-based preprocessing revealed two tight clusters in the left inferior frontal gyrus and left insula, with two additional dipoles within the left middle frontal gyrus. The results of the ICA-based processing were overlapping SOZs detected by the previous invasive study. In summary, the ICA-based preprocessing for the MEG results provided information that was more consistent with other imaging findings, clinical information, and epileptogenic zones identified by SEEG.

This work illustrates the advantages of employing ICA in combination with MNE-Python to enhance the quality of MEG data in patients with RNS devices. This research emphasizes the limitations of tSSS in effectively managing complex artifacts with indeterminate mixtures, potentially resulting in suboptimal artifact removal. Conversely, ICA integrated with MNE-Python offers an automated and potent methodology to detect and eliminate artifact components with uncertain proportions, thereby facilitating more dependable and precise artifact correction. Through the adoption of this methodology, considerable enhancements can be made in the precision and reliability of MEG data, ultimately culminating in more accurate source modeling and improved evaluations for patients utilizing RNS devices.

This study presents a novel approach for analyzing MEG signals derived from epilepsy patients with implanted RNS devices. However, certain limitations are inherent to the methodology and scope of the current investigation. These limitations serve as a foundation for potential avenues of future research, aimed at expanding the applicability and robustness of the proposed method. A primary limitation of this study pertains to the restricted sample size, as only MEG data from two epilepsy patients with identical RNS device models from a single institution were included. This narrow representation of RNS devices may hinder the generalizability of the findings. Future endeavors will encompass a broader cohort. Furthermore, the reliance on contralateral hemisphere channels for establishing a comparative “ground truth” presents an inherent limitation, as it stems from the inability to remove the RNS devices for direct signal validation. While necessitated by practical constraints, this approach introduces an element of uncertainty in the validation process. Alternative validation strategies should include exploration of synthetic signal simulations or innovative signal separation techniques. It is also worth acknowledging that despite the ICA-based processing techniques employed, the MEG signals obtained from channels directly positioned above the RNS devices remain significantly compromised by excessive noise interference. In both patients, the RNS device was implanted close to, or directly over, the seizure onset zone. If feasible, there could be value in implanting the neurostimulator portion of the RNS device away from the SOZ, thereby potentially reducing artifacts in this situation. Nonetheless, this persistent challenge underscores the necessity for continued methodological development. The exploration of refined data acquisition methodologies holds promise for addressing this issue. While this study introduces a method for analyzing MEG signals in the presence of RNS devices, several limitations underscore the necessity for further research. The expansion of the study’s sample size, validation strategies, noise mitigation techniques, and multicenter validation efforts collectively represent essential directions for future investigation. These endeavors are poised to bolster the robustness, reliability, and clinical applicability of the proposed methodology, ultimately contributing to a more comprehensive understanding of MEG signal analysis within the context of RNS devices.

Overall, the present findings suggest that patients with RNS devices can be successfully studied with MEG. Several studies have reported the efficacy of tSSS in removing artifacts from spontaneous MEG recordings in patients with medically interactable epilepsy who have undergone RNS or VNS [14,24,25,26,27]. Nevertheless, there is a lack of research investigating the usefulness of this ICA-based preprocessing technique and assessing its clinical applicability in the context of presurgical epilepsy evaluation. This work represents a novel application of ICA-based preprocessing methods which show promise in removing artifacts while preserving neuronal signals.

## 5. Conclusions

Utilizing ICA-based signal processing beyond the current standard clinical practice, we significantly improved the SNR and clinical utility of the MEG data in patients with RNS devices. However, the SNR in channels directly over the RNS device is still poor and may require modified data acquisition methods in addition to ICA-based preprocessing. This automated method shows promise and future work will include testing in more patients to confirm clinical utility. Utilizing these methods to build upon the current method will allow the fine tuning and improvement of results over time. Overall, patients with MRI-conditional RNS systems should no longer be deemed unable to undergo MEG.

## Figures and Tables

**Table 1:** The results from SNR calculations (mean ± SD (dB)).

	Conventional Method **	ICA-Based Method **	Conventional vs. ICA-Based Method ***
Patient 1	−18.49 ± 1.20	−16.89 ± 0.35	0.14 ± 0.75
Patient 2	−18.22 ± 1.90	−15.66 ± 1.07	−0.61 ± 0.78
Patient 3 *	−1.92 ± 1.61	−2.10 ± 1.47	−0.45 ± 0.24

* Patient 3 did not undergo RNS device implantation; however, in the selection of noise and signal channels, we employed the same criteria as applied to Patient 1, whose RNS device location informed our choice of channels and hemisphere. ** This represents the results from channels above the RNS devices compared to those on the contralateral hemisphere. *** This represents the average results from all channels.

## Data Availability

The data are not publicly available due to patient privacy; however, the de-identified data that support the findings of this study are available from the corresponding author upon reasonable request and formal data use agreement.

## Appendix B

**Table A1:** Individual patients’ information.

	Patient 1	Patient 2	Patient 3
Age (years), Gender	42, F	30, M	67, M
Epilepsy Duration	41 years and 2 months	21 years and 6 months	10 years
Type of Epilepsy	Focal: Lesional Frontal Lobe Epilepsy	Multifocal: Non-Lesional Epilepsy	Focal: Left Mesial Temporal Epilepsy
Seizure Type/Frequency	Focal aware seizures: daily Focal impaired aware seizures: 3 per day Focal to bilateral tonic–clonic seizures: sporadic	Focal unaware seizures: 2 per week Focal to bilateral tonic–clonic seizures: sporadic	Focal impaired aware seizures: 3–4 per week Focal to bilateral tonic–clonic seizures: sporadic
ASM	LCM + LEV + CNB	VPA + LCM +ESL	LCM + BRV
MRI	Left superior and medial frontal lobe cortical dysplasia	Generalized atrophy of the left temporal lobe and left hippocampus. Partial left frontal lobectomy, RNS device in place	Small cortical defect with gliosis in right temporoparieto-occipital lobe; small area encephalomalacia right cuneus
PET	Decreased uptake in left frontal lobe	No definitive discrete areas of abnormal metabolism	Decreased metabolism in left temporal lobe, particularly laterally; frontal lobes hypometabolic as compared to parietal lobes
iEEG	Ictal EEG: Left frontal over FCD; overlapping with SMA Interictal EEG: Left centroparietal breach/slowing *	Ictal EEG: Multifocal; left basal frontal, inferior frontal, anterior temporal, inferior temporal, and insular Interictal EEG: Left frontal polyspikes *	Ictal EEG: Left hippocampus Interictal EEG: Bilateral independent; left >>> right hippocampus
Prior Surgery/ Procedure	SEEG, RNS device placement	Left frontal topectomy and multiple subpial transections, Left partial frontal resection, RNS device placement	None
Surgical Pathology	N/A	Final pathology results unavailable (done at another institution)	N/A

M: Male; F: Female; LCM: Lacosamide; LEV: Levetiracetam; CNB: Cenobamate; VPA: Valproic Acid; ESL: Eslicarbazepine; BRV: Brivaracetam; iEEG: Intracranial EEG; FCD: Focal Cortical Dysplasia; N/A: Not Applicable. * Studies were completed as part of the original pre-surgical workup before RNS device placement and MEG.
